# Timing and extent of finger force enslaving during a dynamic force task cannot be explained by EMG activity patterns

**DOI:** 10.1371/journal.pone.0183145

**Published:** 2017-08-17

**Authors:** Mojtaba Mirakhorlo, Huub Maas, DirkJan H. E. J. Veeger

**Affiliations:** 1 Department of Human Movement Sciences, Faculty of Behavioural and Movement Sciences, Vrije Universiteit, Amsterdam Movement Sciences, Amsterdam, The Netherlands; 2 Department of Biomechanical Engineering, Delft University of Technology, Delft, the Netherlands; Semmelweis Egyetem, HUNGARY

## Abstract

Finger enslaving is defined as the inability of the fingers to move or to produce force independently. Such finger enslaving has predominantly been investigated for isometric force tasks. The aim of this study was to assess whether the extent of force enslaving is dependent on relative finger movements. Ten right-handed subjects (22–30 years) flexed the index finger while counteracting constant resistance forces (4, 6 and 8 N) orthogonal to the fingertip. The other, non-instructed fingers were held in extension. EMG activities of the mm. flexor digitorum superficialis (FDS) and extensor digitorum (ED) in the regions corresponding to the index, middle and ring fingers were measured. Forces exerted by the non-instructed fingers increased substantially (by 0.2 to 1.4 N) with flexion of the index finger, increasing the enslaving effect with respect to the static, pre-movement phase. Such changes in force were found 260–370 ms after the initiation of index flexion. The estimated MCP joint angle of the index finger at which forces exerted by the non-instructed fingers started to increase varied between 4° and 6°. In contrast to the finger forces, no significant changes in EMG activity of the FDS regions corresponding to the non-instructed fingers upon index finger flexion were found. This mismatch between forces and EMG of the non-instructed fingers, as well as the delay in force development are in agreement with connective tissue linkages being slack when the positions of the fingers are similar, but pulled taut when one finger moves relative to the others. Although neural factors cannot be excluded, our results suggest that mechanical connections between muscle-tendon structures were (at least partly) responsible for the observed increase in force enslaving during index finger flexion.

## Introduction

The hand has a complex, mechanically interacting, anatomical structure, most clearly illustrated by the extensor mechanism and the combination of extrinsic and intrinsic muscles [1–3]. This anatomical complexity together with other factors, such as spatial overlap of neurons associated with different fingers in the motor cortex [4], makes it challenging to understand how we control hand movements. One phenomenon in hand motor control is the so-called finger enslaving, defined as the inability of the fingers to move or exert force fully independent of their neighbouring fingers [5–7]. Enslaving has been explained by mechanical and neural connections between muscle (compartments) controlling the fingers [8]. Mechanical connections may be present at the tendon and muscle belly level. Mechanical coupling between the tendons of the extrinsic finger flexors and extensors has been reported previously [3, 5, 9–12]. Intrinsic muscles of the fingers, however, do not appear to be connected to each other. There is also ample proof for force transmission between neighboring muscle bellies [13, 14]. Neurally, enslaving may among others be explained by involuntary co-activation of multiple muscle heads and spatial overlap of motor cortex areas that are responsible for movement of the fingers [5, 8].

Up till now, studies on enslaving focused predominantly on hand function from a kinematic perspective [7, 15, 16] or an isometric force perspective [5, 6, 17, 18]. In the former, subjects were instructed to move specific finger(s) and enslaving was studied by measuring the involuntary movement of other finger(s). In the latter, enslaving was quantified in terms of force by measuring involuntary forces exerted during static finger pressing tasks. In the above studies, the function of the mm. flexor digitorum superficialis (FDS) and mm. flexor digitorum profundus (FDP) is often simplified to isolated finger function by experimentally fixating the wrist and/or to isolated joint function by fixating one or two finger joints.

Enslaving effects during static finger pressing tasks have previously been attributed to neural factors [6, 18–20]. Zatsiorsky et al. studied enslaving by applying force on the different phalanges of the fingers and found similar enslaving effects for the tasks involving predominantly extrinsic (force applied to distal phalange) or predominantly intrinsic muscles (force applied to proximal phalange) [6]. They claimed that enslaving effect should have been different between two mentioned tasks if peripheral connections between tendons of extrinsic muscles had effect on enslaving (because there are no apparent connections between intrinsic muscles in contrast with extrinsic muscles) [6]. In contrast, other studies involving finger movement [15, 21]indicated substantial contributions of mechanical connections. Lang and Schieber [15]showed that the enslaving effect was generally similar during passive (i.e., the finger was flexed and extend by an external force) and active (i.e., voluntary moved) finger flexion movements. As neural factors are not involved during passive movements, they concluded that the mentioned similarity in enslaving indicated a major role for mechanical linkages in enslaving. The contradictory results described above may be related to differences in the tasks studied (static finger pressing and finger movement). Without any finger movement, as in the finger pressing task, mechanical connections may be slack and not able to transmit forces. If a single finger is moved, mechanical connections may experience more strain and, hence, transmit forces. Finger interactions during dynamic tasks, which involve both force exertion and movement (bridging the gap between the two above described experimental approaches) have not yet been investigated and may provide more insight into the different contribution of neural and mechanical connectivity between static finger force and movement tasks.

The aim of this study was to assess if the extent of force enslaving is dependent on relative finger movement. Due to the mechanical properties of connective tissue structures, we hypothesized that the forces applied by the non-instructed fingers will increase as a function of the degree of flexion of the instructed finger, and that the enslaved force will be related to the magnitude of the applied force by that finger. In addition, we hypothesized a short time delay in the build-up of force exerted by the non-instructed fingers. The latter would be in agreement with connective tissue linkages being slack when the position of the fingers are similar, but pulled taut when one finger moves relative to the others. The contribution of neural factors to finger force enslaving will be assessed by comparing the EMG activities of the FDS and extensor digitorum (ED) to the force responses in the non-instructed fingers. If force exertion by the non-instructed fingers is caused by coactivation of muscle fibers, we expected an accompanying increase in EMG of the corresponding FDS muscle regions.

## Methods

### Subjects

Ten right-handed subjects (6 males, 4 females) between 22 to 30 years of age participated. No power calculation was used for sample size calculation, but the number of participants was similar to previous studies [17, 22, 23]. Subjects were master or PhD students, recruited from February till March 2016. Subjects did not report any history of neurological or peripheral disorders of the hand or wrist and did not play a musical instrument. Musicians were excluded, because of a higher than average degree of finger independency [24, 25]. All subjects gave written informed consent according to the regulations established at the Vrije Universiteit Amsterdam. The experiment was performed at the Faculty of Behavioural and Movement Sciences of the Vrije Universiteit Amsterdam, The Netherlands. The Scientific and Ethical Review Board (VCWE) of faculty of human movement and behavioral sciences approved the study protocol.

### Experimental setup

**Fig 1:**
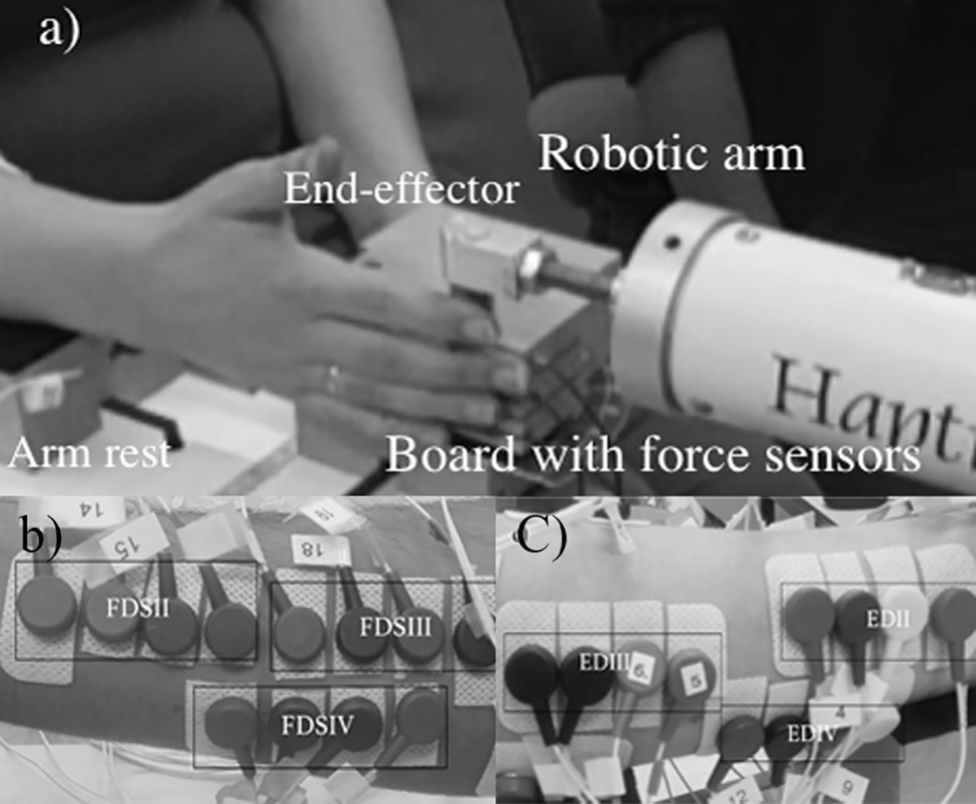
a) Description of experimental set-up showing the board on which the non-instructed fingers were placed and the robotic arm which followed the flexion movement of the index finger. b) Electrode placement on flexor side c) Electrode placement on extensor side.

Subjects were asked to sit on a chair with an adjustable height and to rest their forearm on a horizontal platform leaving the wrist free to move (Fig 1). Their seating position was adjusted such that the elbow was in approximately 90° flexion and the hand in a 90° pronation angle (0° is corresponding to the anatomical position). A wooden board secured to the arm rest was instrumented with three unidirectional force sensors (Futek, Irvine, USA, LSB200, 5 lb). The position of these sensors was adjusted (in two directions: along the finger and medial-lateral) such that the tip of the little, ring and middle fingers was in contact with a narrow beam (width 3 mm) at the center of force sensors. A robotic arm [26] (Haptic Master 2.2, Moog, Nieuw-Vennep, The Netherlands.) equipped with a custom-made end effector was used to provide a resistance force directed along the trajectory of the index finger (Fig 1). Forces exerted by the index finger were measured in three directions.

### EMG

EMG signals were collected in a bipolar configuration with the ground electrode placed on the ulnar styloid, amplified with a 128-channel amplifier and sampled at 2048 samples/s (Refa-136; TMSi, Oldenzaal, The Netherlands). Electrode placement sites were shaved and cleaned with alcohol. Muscle regions of FDS and ED were palpated for each subject individually. Multiple electrodes were used for each muscle region in order to cover a broader area, as inter-individual differences in muscle structure hamper the identification of each correspondent muscle region of each finger with palpation only. Four electrodes (KendallTM H69P Cloth Electrodes, Covidien, Zaltbommel, The Netherlands) were placed on each of the regions corresponding to the index (II), middle (III) and ring (IV) fingers for both ED and FDS muscles (.1b & c). Thus for each muscle region, there were six possible bipolar combinations. Muscle activities corresponding to the little finger were not measured in this study as we did not expect to find enslaved forces [6] or substantial EMGs for the little finger. Electrodes were linearly placed at an inter-electrode distance of approximately 2 cm (Fig 1B & 1C). To check the validity of placement for each muscle region, subjects were instructed to flex the related finger freely with real-time display of the EMG signals on a monitor.

To record the maximal EMG of the extensor regions, subjects were asked to maximally extend their finger while it was held flexed by the experimenter. Each MVC trial was repeated three times. EMGs were averaged over the full three seconds during each repetition; and the maximum value was selected for normalization. For the extensor EMGs, the electrode pair yielding the highest amplitude during the MVC tests was selected.

To find the best representative signal for the different FDS muscle regions (index, middle and ring finger), a ramp protocol was performed. With the index finger placed against the fixed robotic arm and the other fingers on the board, subjects were asked to gradually increase flexion force to a submaximal level (around one third of maximal force) for each finger individually during which both EMGs and forces were measured. Each subject repeated this task three times. The ratio of each FDS region and the corresponding ED muscle region was calculated for each every electrode combination. The electrode combination for which the highest ratio was found during force exertion of the target finger and the lowest ratio during force exertion of the other fingers was selected for further analysis.

### Experimental protocol

Subjects were asked to build up forces with their index finger (instructed finger) to predefined levels of 4, 6 and 8 N against the fixed end-effector of the robotic arm and maintain this force for at least one second (static phase). During this phase, they were given real-time visual feedback about the applied force level. Force buildup was followed by a phase in which the end-effector was programmed to follow the path of the index finger, producing a resistance force at the same predefined levels (dynamic phase). The robotic arm was programmed such that it exerted force in the same direction as the velocity of the movement of the tip of the index finger. The subjects were asked to flex only the MCP joint of the index finger from an extended position (i.e., metacarpophalangeal, MCP, proximal interphalangeal, PIP, and distal interphalangeal, DIP, joints at 0°.) to a more flexed position (approximately 45°). The PIP and DIP joints were also free to move, but subjects were instructed to minimize this. The other, non-instructed fingers (middle, ring and little) were resting against the board with force sensors (Fig 1A). Subjects were asked to not pay attention to the non-instructed fingers. Fingertip forces were recorded simultaneously with EMG signals, as well as the end point trajectory of the index finger. The robotic arm and the EMG data recording system were synchronised using a start-stop pulse signal to both devices. Each subject repeated the task until at least two trials with an average end-effector speed of around 3 cm/s were recorded. This average speed was selected, as in pilot measurements it corresponded closely to the self-selected speed of finger flexion movement, which was a comfortable speed (neither too slow nor too fast) according to the feedback of the participants.

### Data analysis and statistics

EMG signals were high pass filtered at 20Hz using a fifth order, zero-lag Butterworth filter. Subsequently, signals were rectified on basis of the Hilbert transformation and, then, low pass filtered at 2 Hz also using a fifth order, zero-lag Butterworth filter. Mean EMG activities and forces during the static phase were calculated. During the dynamic phase, EMG activities and forces at the end of the movement were used for further analysis. Force signals of all fingers and position data of the index finger were low pass filtered at 10Hz using a third order, zero-lag Butterworth filter. The forces exerted by the subjects in the resting position were adjusted to zero.

**Fig 2:**
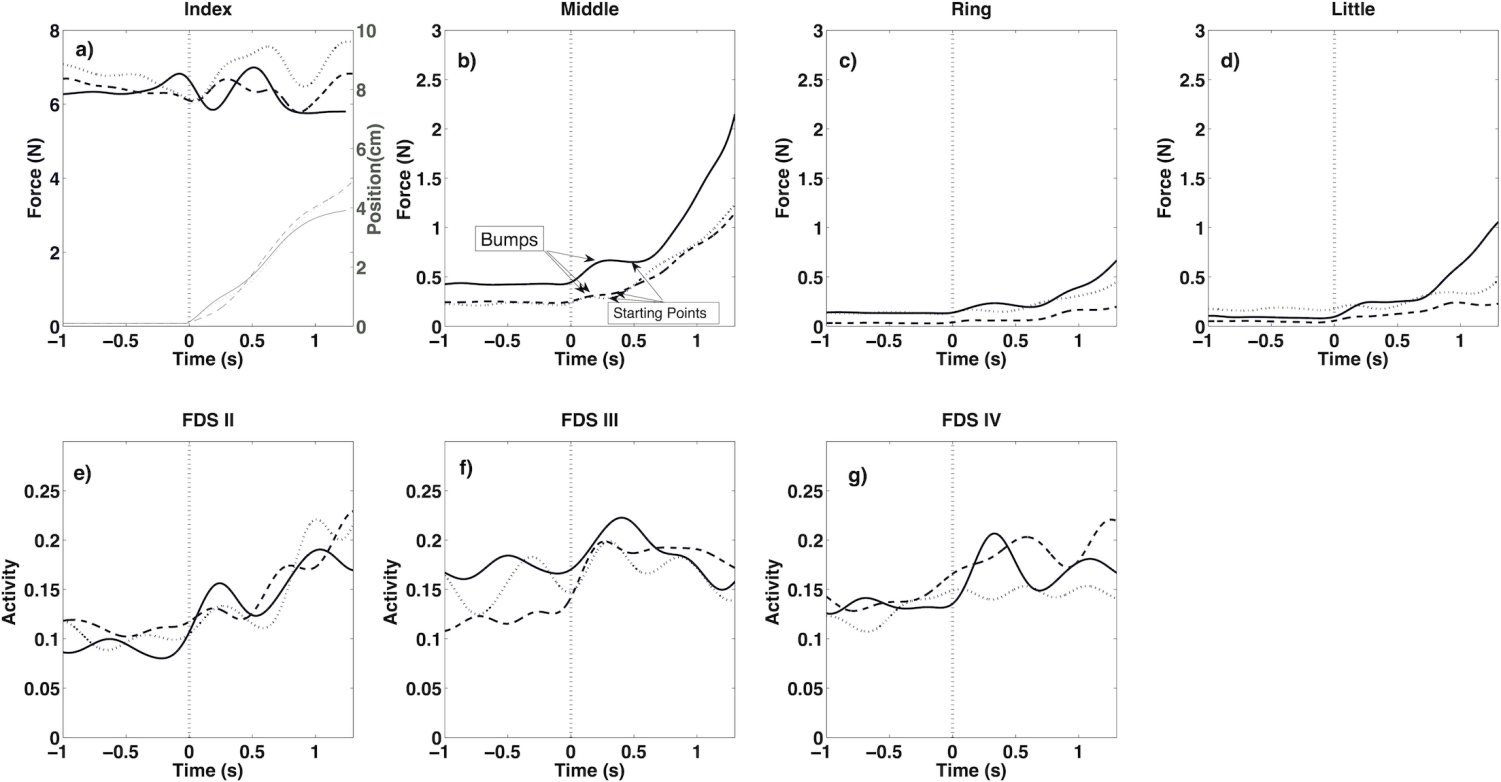
Force (thick lines) and position (thin lines) of target finger, index (a), forces of non-instructed fingers (b-d) and EMGs of FDS muscle for related fingers (e-g) during static phase (Time: -1 to 0) and dynamic phase (Time: 0 to 1.35) (see the first paragraph of “Experimental protocol” section). Data presented in this figure were related to one representative subject during 3 trials (solid, dashed and dotted lines). Vertical dashed line indicates the start of movement.

The instant of force buildup was defined as an increase in force of more than 1% of the force during the static phase, the latter calculated as the mean of one second prior to the start of index finger movement. The time between the start of index finger movement and the instant of force buildup was defined as the time delay. Little bumps (small increases in force followed immediately by a force decrease) in the pattern of forces (Fig 2B) were ignored, because they were the result of perturbations caused by the start of the movement and instability of the robotic arm. We programmed the robotic arm in a way that it applied a resistance force in the direction of movement velocity. Noise in the position signals used for feedback caused an unintendedly applied force and a sudden movement at the initiation of the dynamic phase of the task. This noise was very low relative to the index finger movement and, therefore, did not affect the applied force by the robotic arm. Due to the instability of the robotic arm, there is a sudden drop in index finger force at the beginning of the movement (Fig 2A). The drop induces a small perturbation to the subject’s hand causing the bumps in the non-instructed finger forces. The results of the representative subject clearly show that the more accented the force drop in the index finger force (thick solid line) the higher the force bumps of the non-instructed fingers. The points that were considered to be the start of the increase in the middle finger force are indicated in Fig 2B.

The force enslaving effect (EE) of each finger was calculated, for both static and dynamic phases. For the dynamic phase, EE was calculated, as the ratio of the force exerted by the non-instructed finger at the end of index finger movement to the force of the index finger at the same time point. For the static phase, averaged forces during 1-second prior to start of movement were used to calculate the EE. In addition, the difference in EE between static and dynamic phase was calculated (ΔEE).

$$EE\%=\frac{{Force}_{non-instructed}}{{Force}_{index}}\times 100\%$$

Velocity of the fingertip (robotic arm end-effector) was calculated as the first derivative of the position signal. The MCP joint flexion was estimated based on the length of the subject’s index fingers and the position of the index finger tip, assuming DIP and PIP joint angles were kept at zero degrees.

A one-way ANOVA was used to compare the mean velocities of the index fingertip for the different force resistance conditions. For the instructed index finger, two-way repeated measures ANOVAs (factors: static-dynamic phase and resistance force level) were used to test for changes in FDS, ED muscle activities and force. Two-way repeated measures ANOVAs were used to analyze effects of resistance forces levels and differences between non-instructed fingers on the time delays, corresponding index MCP angles and ΔEE. In case of significant interactions, a post-hoc t-tests with Bonferroni correction was performed. Three-way repeated measures ANOVAs (factors: finger, static-dynamic phase and resistance force level) were used to test for changes in FDS, ED muscle activities, force and EE in the non-instructed fingers. All analyses were performed in Matlab (2017a, Mathworks, Natick, USA).

## Results

### Index velocity and MCP angle of the index finger

The mean velocity of the index finger tip was 3.1±0.4, 3.0±0.3 and 3.0±0.4 cm/s for the 4, 6 and 8N resistance force levels, respectively. These velocities were not significantly different between force levels [F (2, 29) = 1.04, p = 0.367]. The estimated maximum MCP flexion of the index finger was MCP 37°±13°.

### Time delays and corresponding index MCP angles

**Fig 3:**
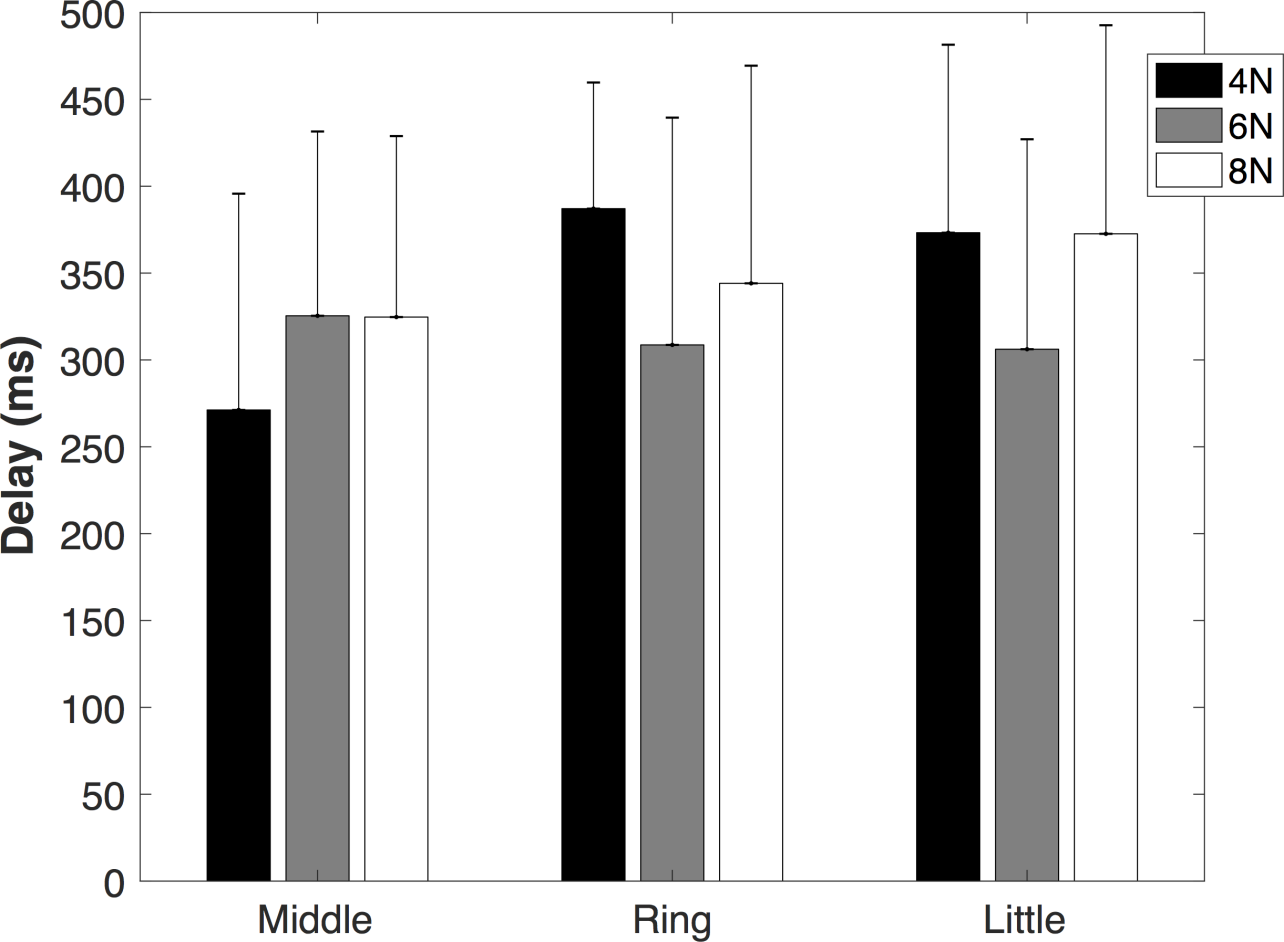
Time delays of non-instructed (middle, ring and little) finger forces during different resistance forces (4N, 6N and 8N).

The non-instructed fingers (middle, ring and little) started to exert forces with a delay relative to the start of the movement of the index finger. The delay ranged from 260 to 370 ms depending on the finger and force resistance conditions (Fig 3). The delays were not significantly dependent on resistance force levels [F (2,18) = 0.864 p = 0.438] and not different between fingers [F (2,18) = 1.409 p = 0.27]. Moreover, there was no significant interaction between factors (force level and finger) [F (4,36) = 2.29 p = 0.079]. The estimated index MCP joint angle at which the non-instructed fingers started to exert forces was 4°±2°, 5°±2° and 6°±3° for middle, ring and little fingers respectively. Note that the mentioned numbers indicate the average across all force resistance conditions. These MCP angles were not significantly affected by changes in resistance force levels [F (2,18) = 2.281 p = 0.131], not different between fingers [F (2,18) = 3.376 p = 0.057]; also no significant interaction was found [F (4,36) = 2.223 p = 0.086].

### EMG activities and finger forces

The activity of the index finger flexor, FDSII, changed significantly for 3.7%, 4.9% and 5.8% from the static to the dynamic phase for 4, 6 and 8 N resistance forces, respectively (F (1,9) = 5.86 p = 0.039). ANOVA indicated no significant differences in activity of the index finger extensor, EDII, (F (1,9) = 3.11 p = 0.111) and force exerted by the index finger (F (1,9) = 2.27 p = 0.166) between the static and dynamic phase of the task.

**Fig 4:**
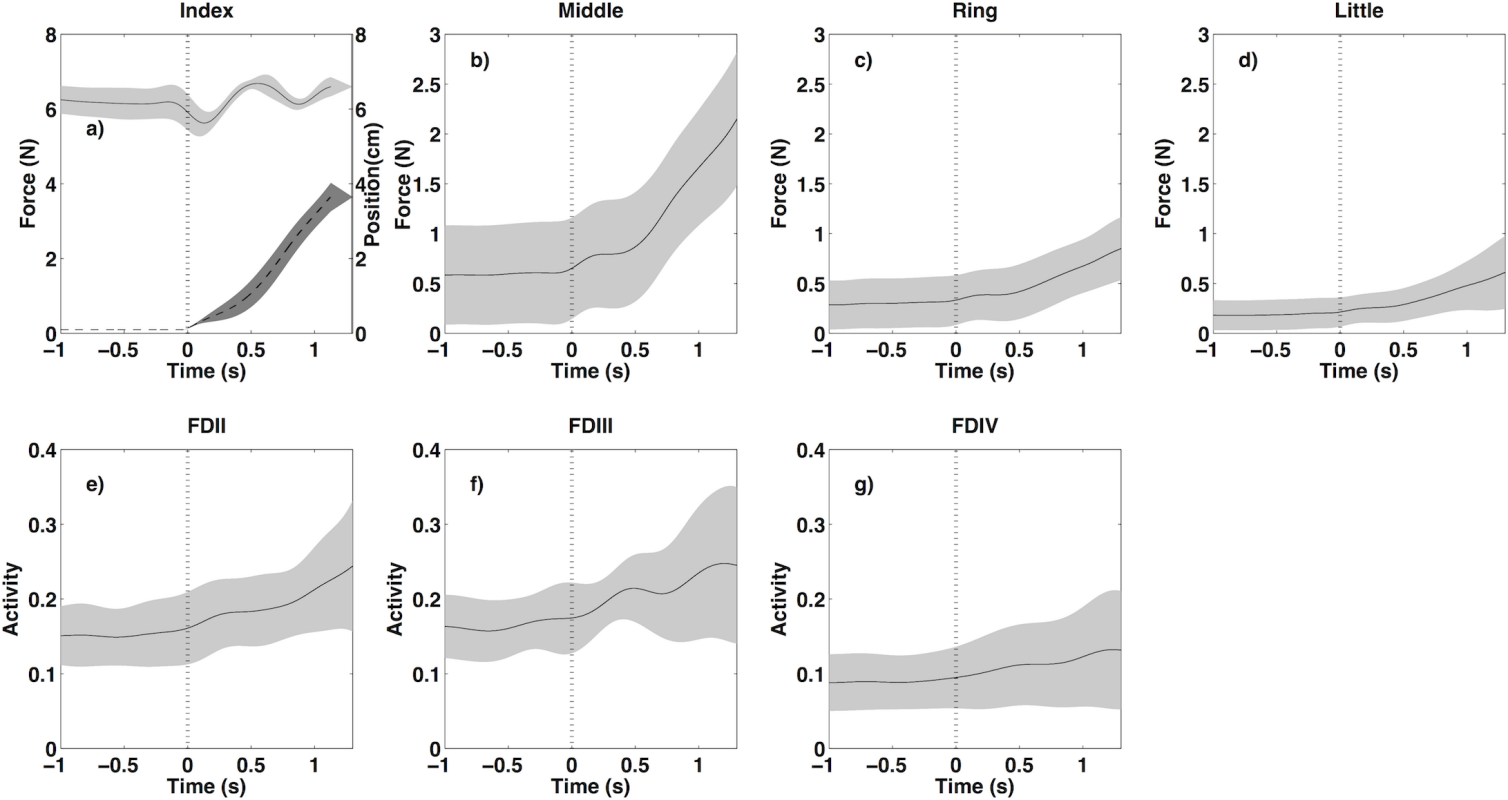
Finger forces (a-d) and FDS EMG activity patterns (e-g) averaged across all over subjects for 6 N resistance force. For index finger (a), its position (darker surface color with dashed line) was also plotted. Vertical dashed lines indicate the start of index finger movement.

The activity of the non-instructed FDS regions (III and IV) remained almost constant during the task for all resistant forces (Table 1, ANOVA indicated no significant changes, see Table 2), while there was an increase in the related fingers forces (Tables 1 & 2, Fig 4). No significant changes in EMG activity of ED muscle were found (Tables 1 & 2). Note that the activity of little finger FDS (V) was not recorded in our experiment. Activity of FDS regions and forces of non-instructed fingers increased significantly as the resistance force increased (Tables 1 & 2).

**Table 1:** EMG activity of FDS and ED muscle regions and forces exerted by the fingers during the static and dynamic phases of the task (means ± SD).

Resistance Force	Finger	Activity FDS (%MVC)			Activity ED (%MVC)			Force (N)		
		Static Phase^†^^+^ (%)	Dynamic Phase^#^ (%)	Difference (%)	Static Phase^†^ (%)	Dynamic Phase^#^ (%)	Difference (%)	Static Phase^†^ (N)	Dynamic Phase^#^ (N)	Difference (N)
4N	Index	10.9±3.4	15.6±5.3	3.7±2.9	6.9±3.7	6.8±3.9	-0.9±0.9	4.3±0.1	4.3±0.21	-0.0±0.6
	Middle	13.2±5.7	15.1±7.6	1.9±4.1	5.3±4.1	4.9±4	0.7±1.2	0.3±0.2	1.3±0.41	0.86±0.3
	Ring	6.8±3.6	7.2±3.6	0.7±0.9	4.7±3.4	4.9±3.9	0.7±0.7	0.15±0.2	0.43±0.2	0.27±0.1
	Little	*	*	*	*	*	*	0.1±0.0	0.31±0.1	0.21±0.2
6N	Index	13.6±3.8	18.6±7.7	4.9±3.3	7.3±2.7	10.0±6.1	2.7±4	6.2±0.4	6.5±0.2	0.4±0.5
	Middle	16.3±5.8	21.5±15.3	5.2±7.3	4.4±3.2	5.7±4.2	1.5±1.7	0.6±0.5	1.8±0.6	1.2±0.4
	Ring	8.0±3.6	10.9±7.8	2.9±5.3	4.4±2.6	7.5±7.3	3.1±5.4	0.3±0.2	0.7±0.3	0.4±0.2
	Little	*	*	*	*	*	*	0.2±0.1	0.5±0.3	0.26±0.3
8N	Index	19.1±6.7	23.9±7.8	4.8±6.8	9.5±5.7	12±7.8	3.3±4.6	8.1±0.1	8.2±0.4	-0.1±0.4
	Middle	20.9±8	25.7±15.9	4.8±10.4	5.7±3.1	7.1±5.1	1.4±3.2	0.75±0.6	2.1±1	1.4±0.9
	Ring	11.9±10.2	12.2±6.54'	0.2±5.2	6.4±5.9	8.0±6.2	2.2±4.0	0.34±0.3	0.8±0.4	0.4±0.2
	Little	*	*	*	*	*	*	0.2±0.2	0.6±0.3	0.3±0.2

* EMGs of little finger was not measured

† Averaged during the phase

# The value at the end of phase

**Table 2:** p and F values (df1 = factors degree of freedom, df2 = errors degree of freedom) of three-way repeated measures ANOVAs applied to statistically analyze changes in either finger forces, FDS and ED activity of non-instructed fingers when switching between phases (static and dynamic) phases as a function of the amplitude of the resistance force.

Factors and interactions	Activity FDS		Activity ED		Force	
	F (df1, df2)	p	F (df1, df2)	p	F (df1, df2)	p
Finger	14.63 (1,9)	0.004	0.24 (1,9)	0.632	48.61 (2,18)	<0.001
Resistance force level	4.93 (2,18)	0.02	2.33 (2,18)	0.126	11.95 (2,18)	<0.001
Static-dynamic phase	1.532 (1,9)	0.247	2.47 (1,9)	0.150	76.22 (1,9)	<0.001
Finger × Force level	1.466 (2,18)	0.257	1.10 (2,18)	0.353	5.00 (4,36)	0.003
Finger × Phase	0.611 (1,9)	0.454	0.91 (1,9)	0.364	38.91 (2,18)	<0.001
Force level × Phase	2.384 (2,18)	0.121	2.37 (2,18)	0.122	5.23 (2,18)	0.016
All three factors	1.811 (2,18)	0.192	0.99 (2,18)	0.389	0.75 (4,36)	0.563

### Additional force enslaving

The highest EEs were observed for the middle finger (Table 3). The change in EE due to the shift from the static to dynamic phase of index finger was highest for the middle finger during all three conditions: 24.7%, 19.4% and 16.5% for the 4, 6 and 8 N resistance forces levels. The changes in EE for the ring finger and little finger were relatively small (less than 6% for all conditions). Post-hoc comparison showed that the change of EE in middle finger was significantly higher than that of the ring and little fingers (p< 0.001 for both cases). There was no significant difference between the little and ring finger (p = 0.280).

**Table 3:** Force enslaving effect (EE), ratio of non-instructed finger forces to index finger force, before the movement (static phase), at endpoint of the movement (dynamic phase) and the change between them for three force resistances.

Resistance Force	Non-instructed Finger	Enslaving Effect %		
		Static phase	Dynamic phase	Change (Additional force enslaving)
4N	Middle	6.5±5	31.2±9.7	24.7±8.6
	Ring	3.6±3.8	10.0±4.3	6.4±1.8
	Little	2.4±2.3	7.1±3.2	4.7±2.6
6N	Middle	9.5±5.4	28.9±9.5	19.4±6.9
	Ring	4.5±3.7	10.4±4.4	6.1±2.4
	Little	2.8±2.2	7.9±4.3	5.1±3.8
8N	Middle	9.2±5.3	25.7±13.3	16.5±9.1
	Ring	4.2±4.0	10.1±4.5	6.0±2.3
	Little	3.2±3.2	7.5±4.4	4.3±3.2

Three-way repeated measures ANOVAs results for: Finger [F (2,18) = 51.974, p<0.001], Force level [F (2,18) = 3.756, p = 0.043], Phase: [F (1,9) = 76.061, p<0.001], Finger × Force level [F (4,36) = 0.796, p = 0.536], Finger ×Phase [F(2,18) = 36.342, p<0.001] and Force level × Phase [F(2,18) = 3.578, p = 0.049]

## Discussion

The aim of this study was to investigate if the extent of force enslaving is dependent on movement of fingers relative to each other. We found that non-instructed finger forces increased during isotonic flexion of the instructed index finger, but with a substantial time delay. Our data showed also that the EMG activities in FDS regions corresponding to the non-instructed fingers did not change significantly upon flexion of the index finger.

The extent of force enslaving was higher during index finger flexion (dynamic phase) than during the static phase of the task, independent of force level. Considering that there were no significant changes in EMG activities of the non-instructed fingers, these results indicate that the increase in the EE during movement should most likely be attributed to the relative movement of the instructed index finger, most likely mediated by the effects of mechanical connections between muscle compartments and tendons. The delay in force response of the non-instructed fingers further supports a role of mechanical connections in the observed enslaving. This delay may be explained by intertendinous or intermuscular connections initially being slack and pulled taut as a result of flexion of the index finger, which involves displacement of the related tendon in proximal direction and shortening of the in series muscle fibers. The delayed force transmission is in agreement with the mechanical properties of connective tissue linkages (i.e., the slack length and the non-linear stress-strain characteristics). We found no significant differences in the velocity of movement or in the delays and their corresponding estimated index MCP joint angles between force resistance conditions. This indicates that the position in which non-instructed finger forces start to increase was not affected by changes in the resistance force, but apparently depends on the relative position of the index finger. If enslaving were due to co-activation as a result of diverging neural commands, a more instantaneous increase in non-instructed finger forces would have been expected.

To our knowledge, no other studies investigating finger force enslaving with a similar task as in our experiment have been reported. However, in agreement with our findings Van den Noort et al. [21] found that the index finger could flex independently, i.e. without movements of the non-instructed fingers, for some range. Also, Li et al. [16] reported delays between movement of instructed and non-instructed fingers during isolated distal inter-phalangeal joint flexion while restricting other joints. They attributed the delay to the longer latencies of the neural pathways involved in excitation of FDP regions of the enslaved fingers arguing that the FDP tendons are fully separated.

Despite our conclusion that mechanical factors likely play a major role for the increase of enslaving in response to finger movment (i.e., from the static to the dynamic phase), we cannot exclude the contribution of neural factors. Other muscles (i.e. the intrinsic hand muscles and FDP) that were not measured in the present study may also be responsible for the force changes in the non-instructed fingers. Intrinsic muscles were previously suggested to be involved in force enslaving [6]. However, it has also been concluded that during large finger movements, such as in the present study, the intrinsic muscles are less active than the extrinsic muscles [2]. Also, during hand and finger motor tasks, the levels and patterns of activation for FDP and FDS are similar [27, 28]. As our results on FDS indicated no neural divergence during index finger movements, this was most likely also not the case for FDP. For future studies, ideally the activity of all involved muscles needs to be measured. In combination with a musculoskeletal model of the hand and wrist incorporating all involved muscles (e.g. [29]), the contribution of neural and mechanical connectivity in finger enslaving can be unraveled.

Although the resistance force applied to the instructed index finger was constant, the activity level of the FDS in index region increased during movement (Table 1). This may be explained by the change from isometric to concentric muscle contraction (i.e. higher levels of muscle activity are required to maintain a certain force during concentric muscle conditions [30] and by muscle fibers producing force at lower lengths, which yields less force if on the ascending limb of the length-force curve [31]. Alternatively, due changes in length and relative position of the FDS compartment linked to the index finger more force may be ‘leaking’ (transmitted) to the tendons of the non-instructed fingers [32, 33]. As a consequence, more activity in FDSII is required to exert a constant force at the tip of the index finger.

Previous studies that focused on static tasks concluded that neural factors are the dominant mechanism behind the enslaving phenomenon [6, 20]. We found a significant increase in non-instructed finger forces and corresponding FDS EMGs during the static phase as a function of resistance force level (Tables 1 & 2), which supports the role of neural factors in the static phase of the task. However, during the dynamic phase the evidence agreed with a significant role of inter-connections of tendons and muscle bellies (see above). This is in line with previous work [15] concluding that mechanical connections between muscle-tendon units play a major role during finger movements. In a similar experiment to our study, also an increasing force in the non-instructed finger was observed upon flexion of a neighbouring target finger [23]. In contrast to our study, these authors could not quantify the enslaving effect in terms of forces as the target finger moved freely without resistance force. The EEs of the middle finger the static phase calculated in this study (6.5–8.5%, Table 3) were slightly higher than the results of a previous study [22] which reported 3.5% EE for the middle finger during submaximal index finger force exertion (25% MVC). Additional force enslaving, the change in EE as result of shift in dynamic and static phase, seems to be induced by the movement and their values were significantly higher for the neighboring middle finger (17 to 24%) than for the two other non-instructed fingers (Table 3). This can also be interpreted in favor of mechanical linkages role in enslaving.

Most previous studies investigating enslaving constrained the wrist during their experiments. In the current study, we did not fix the wrist and, thus, it had to be stabilized by wrist flexors (including FDS) and extensors (including ED). As a consequence, application of an external force to the finger, requiring a finger flexion moment, also requires wrist flexion moment, which may be produced by FDS and FDP. Imposing restriction to wrist joint movement causes simplification of FDS/FDP function and limits the mechanical interaction between hand and wrist.

This study had several limitations. The EMG activity of only two muscles was measured, while several other muscles (such as FDP and intrinsic) contribute to the task studied. Using fine wire EMG would have been helpful to detect FDS activities more precisely. However, we used multiple electrodes to cover a broader area to find electrode pairs that as uniquely as possible represent the different muscle regions. Finally, maximum static flexion forces of each of the fingers were not assessed. Based on previous studies [34], our force levels correspond to approximately 8%, 12% and 16% of MVC.

In conclusion, increased force enslaving of the middle finger in response to index finger movement cannot be explained by changes in neural drive to the corresponding muscle regions of FDS and ED. This suggests that for the task studied, mechanical connections between muscle regions and/or tendons are (at least partly) responsible for limiting finger independency.
